# Comparative Transcriptome Sequencing of Taro Corm Development With a Focus on the Starch and Sucrose Metabolism Pathway

**DOI:** 10.3389/fgene.2021.771081

**Published:** 2021-10-22

**Authors:** Weiqing Dong, Fanglian He, Huiping Jiang, Lili Liu, Zuyang Qiu

**Affiliations:** ^1^ Biotechnology Research Institute, Guangxi Academy of Agricultural Sciences, Nanning, China; ^2^ Lipu Municipal Bureau of Agriculture and Rural Affairs, Lipu, China

**Keywords:** carbohydrates, gene expression, small RNA, corm development, taro (*Colocasia esculenta* L. Schott)

## Abstract

Taro (*Colocasia esculenta*) is an important tuber crop and staple food. Taro corms have higher nutritional value and starch contents as compared to most of the other root/tuber crops. However, the growth and development of the taro rhizome have not been critically examined in terms of transcriptomic signatures in general or specific to carbohydrates (starch and sucrose) accumulation. In current study, we have conducted a comprehensive survey of transcripts in taro corms aged 1, 2, 3, 4, 5, and 8 months. In this context, we have employed a whole transcriptome sequencing approach for identification of mRNAs, CircRNAs, and miRNAs in corms and performed functional enrichment analysis of the screened differentially expressed RNAs. A total of 11,203 mRNAs, 245 CircRNAs, and 299 miRNAs were obtained from six developmental stages. The mRNAs included 139 DEGs associated with 24 important enzymes of starch and sucrose metabolism. The expression of genes encoding key enzymes of starch and sucrose metabolism pathway (GBSS, AGPase, UGPase, SP, SSS, βFRUCT and SuSy) demonstrated significant variations at the stage of 4 months (S4). A total of 191 CircRNAs were differentially expressed between the studied comparisons of growth stages and 99 of these were associated with those miRNA (or target genes) that were enriched in starch and sucrose metabolism pathway. We also identified 205 miRNAs including 46 miRNAs targeting DEGs enriched in starch and sucrose biosynthesis pathway. The results of current study provide valuable resources for future exploration of the molecular mechanisms involved in the starch properties of Taro.

## Introduction

Taro (*Colocasia esculenta*) has a long cultivation history and is an important nutritional resource in the world, particularly in China. China ranked third with 18% of the global production (1,908,830 tons) (FAOSTAT 2021) and China ranked first in taro export (417.18 million US$ in 2018) (Otekunrin et al., 2021). Taro corms have higher nutritional value as compared to most of the other root/tuber crops. It has been shown that both leaf and corm are rich sources of good-quality protein as well as nutrients including calcium, potassium, and phosphorus (Temesgen and Retta, 2015). The edible part of taro is the corm, which is a source of protein, carbohydrate, fat, crude fiber, vitamin C, thiamin, riboflavin, and niacin (Temesgen and Retta, 2015). Starch is the most important component of taro corms (Njintang et al., 2007). The carbohydrate content of taro corms is almost double of that of potato with an energy of 135 kcal/100 g. The protein content is also 11% higher than yam, cassava, and sweet potato (Njintang et al., 2007). Due to the presence of such a rich content of nutrients, it is important to understand the genetic basis of the nutrient composition. Particularly, the highest starch content in taro corms calls for a detailed understanding of the transcriptomic signatures that might regulate the related pathways (Otekunrin et al., 2021). It has been reported that the growth of the main plant is completed in three phases i.e., phase I (1 to 6–8 weeks), II (8–24 weeks), and III (25–40/46 weeks). The first phases last for about 6–8 weeks. The first 2 weeks result in a decrease in dry matter content of the corm followed by a steady increase in dry matter contents till the 8th week after planting (Sivan, 1979). The early growth phase i.e., phase I is essential for plant survival and early accumulation of dry matter and nutritive components. During phase II, dry matter accumulates rapidly. This trend is further extended till phase III (Sivan, 1979; Tumuhimbise et al., 2009). In this regard, the growth and development of the taro corm have not been critically examined in terms of transcriptomic signatures in general or specific to carbohydrates (starch and sucrose) accumulation.

The carbohydrates are mainly biosynthesized through the starch and sucrose metabolism pathway and the pathways that are present both up- and downstream e.g., glycolysis/gluconeogenesis pathway, and amino sugar and nucleotide sugar metabolism (Preiss, 1982; MacRae and Lunn, 2006). The major enzymes that take part in different steps of these pathways are sucrose synthase, invertase, sucrose phosphate synthase, ADPG pyrophosphorylase, starch synthase, starch branching enzyme, starch debranching enzymes, and starch phosphorylase (Preiss, 1982; MacRae and Lunn, 2006; Gao et al., 2018). Starch is synthesized in plastids (chloroplasts) in leaves. Of the starch synthesizing enzymes, three are the most important. The first enzyme, starch synthase, takes part in the elongation of non-reducing ends of glucose chains. The second enzyme i.e., the branching enzyme, synthesizes branches from existing chains through glucanotransferase reactions. While the third type of enzyme (debranching enzymes) hydrolyzes some of the branches again. These three steps are simultaneous and interdependent processes (Gao et al., 2018). Our current understanding of these biosynthetic enzymes is very much advanced in different plant species. Yet, the identification and functional validation of starch biosynthesis-related genes in taro remain to be studied. An earlier study on taro leaves used the transcriptome sequencing (mRNA) approach to identify the putative genes involved in starch biosynthesis in taro and reported 26 genes e.g., starch branching enzyme A, soluble starch synthase I, II, and UDP-glucose dehydrogenase (Liu et al., 2015). However, this study was limited to leaves only and didn’t explore the main edible part of taro i.e., corm, which is considered the main source of starch. Another study reported the identification and cloning of an ADP-glucose pyrophosphorylase and confirmed that its higher expression is positively correlated with higher starch contents in taro corms (Li et al., 2016). However, the knowledge on the regulation of this and other starch and sucrose synthesis-related genes in the early growth period is still scarce.

Recent developments in genomics have resulted in an increased understanding of the genome as well as specific pathways in different crop plants. In this regard, the release of a high-quality genome sequence of taro is an important step (W. Li et al., 2016). Concomitant developments in sequencing approaches are already helping researchers to understand how different traits are regulated in taro. For example, transcriptome sequencing revealed the possible mechanism of purple pigment formation (He et al., 2021) and the development of EST-SSR (You et al., 2015), and SSR markers (Wang et al., 2017) in taro. Other studies using deep sequencing (Illumina Hiseq 2000) of the taro transcriptome have explored the major metabolic pathways of starch synthesis. This study greatly helped to identify the mRNAs (and respective genes) that are expressed in taro corm for the biosynthesis of starch [See Table 4 in Liu et al., 2015]. Though this study reported the major genes responsible for starch biosynthesis, but how the expression of these genes is modulated during corm development is not known. Additionally, the role of miRNAs and CircRNAs in corm development is yet to be elaborated. Since earlier studies have reported that miRNAs can modulate the stability of starch biosynthesizing enzymes in wheat (Goswami et al., 2014) and form a complex network in maize (Zhang et al., 2019) to regulate starch biosynthesis. Thus, the role of miRNAs in corm development and starch and sucrose metabolism could be expected. This expectation is based on the earlier report in cassava that miRNAs effect the expression of genes involved in plant development, starch biosynthesis, and responses to the environmental stresses (Panigrahi et al., 2021). Since, CircRNAs act as miRNA sponge to regulate target gene expression by inhibiting miRNA activity. Furthermore, one CircRNA can regulate multiple miRNAs (Hansen et al., 2013). Similarly, the role of CircRNAs have not been explored yet for their role in starch and sucrose biosynthesis pathway in taro (Hansen et al., 2013). Through the whole transcriptome sequencing approach (mRNA, CircRNA, and miRNA), we have conducted a comprehensive survey of transcripts in taro corms aged 1, 2, 3, 4, 5, and 8 months. We specifically focused on the starch and sucrose metabolism pathway and the two pathways present up and downstream i.e., amino sugar and nucleotide sugar metabolism, and glycolysis/gluconeogenesis pathways, respectively.

## Materials and Methods

### Plant Material

Taro (*Colocasia esculenta* L. Schott) variety “Guiyu No. 2” was grown in field conditions in Guangxi Academy of Agricultural Sciences, Nanning, Guangxi, China in March 5, 2020 following the agronomic practices and growing conditions recommend by Onwueme et al. (Onwueme, 1999). One, two, three, four, five, and eight-months old taro corms were harvested separately, washed thoroughly with running water and then with distilled water. Samples were immediately frozen in liquid nitrogen, and stored in −80°C refrigerator until processed for RNA extraction. Three samples from three different plants were harvested at each sampling time.

### RNA Extraction, Library Preparation, RNA Sequencing, Read Mapping, and Transcriptome Assembly

Total RNA was extracted from the 18 corms (triplicate samples of S1-S6) using TRIzol Reagent (Invitrogen, Carlsbad, CA, United States) according to the manufacturer’s instructions. The quality and integrity of the extracted RNAs were tested with Agilent 2,100 Bioanalyzer (Agilent Technologies, United States) and NanoDrop 2000 spectrophotometer (Thermo Scientific, United States), respectively.

The libraries for three RNA types i.e., mRNA, miRNA and CircRNA, were prepared as follows. First, we removed the ribosomal RNA by using the Ribo-Zero Plant Kit (Illumina, San Diego, CA, United States) according to the manufacturer’s instructions. After the removal of rRNA, the libraries were preparing using TruSeq Stranded Total RNA Library Prep kit according to the manufacturer’s protocol. For each sample, 5 µg total RNA was used. For small RNA libraries preparation for each taro sample and replicate, 3 µg of the total RNA was used and processed by using Truseq Small RNA sample prep Kit (Illumina, United States) according to the manufacturer’s instructions. The libraries were then quantified in a Fluorometer (TBS-380, Turner Biosystem, United States) followed by sequencing on an Illumina HiSeq platform.

For validation of RNA-seq data, qRT-PCR was performed using qtower3 G (Jena Analysis, Germany) system. One microgram RNA was used for the first strand cDNA synthesis using MonScript™ RTIII All-in-One Mix with dsDNase. The QuantiNova SYBR Green RT-PCR Kit was used for qRT-PCR reaction. The reactions were carried out by using gene specific primers (Table 1). The 2^−ΔΔCt^ method was used to analyze relative gene expression (Livak and Schmittgen, 2001). The *Taro-actin* gene was used as an internal control (Lekshmi et al., 2020).

**TABLE 1:** List of primers used for qRT-PCR analysis.

Gene ID		Primer sequence
*Taro-Actin*	Forward	CCT​TCG​TCT​TGA​TCT​GGC​AG
	Reverse	AGA​TGA​GTT​GGT​CTT​CGC​AGT​C
*Colocasia_esculenta_newGene_13195* (beta-fructofuranosidase)	Forward	CCC​TTG​AAC​AAT​GCT​ACC​CC
	Reverse	CAT​CTT​AGC​CAC​CTC​CTC​GTC
*Colocasia_esculenta_newGene_17987* (alpha-amylase)	Forward	GAC​ATC​CAC​AGC​CGT​TCA​GC
	Reverse	TTG​CCA​GAG​TCC​ACT​CCC​TC
*Colocasia_esculenta_newGene_2769.1* (beta-amylase)	Forward	CAT​TCT​TTT​GTG​ATG​GAG​GGG
	Reverse	GCA​TGG​CTG​GCT​GTC​TTG​TA
*Colocasia_esculenta_newGene_46372.1* (glucose-6-phosphate isomerase)	Forward	GCA​GAA​TGT​GGA​AAA​GGC​AGA​C
	Reverse	GAA​GAA​ATC​CAT​TCC​CTC​AGT​GTT
*Colocasia_esculenta_newGene_50233.1* (UTP--glucose-1-phosphate uridylyltransferase)	Forward	ATG​TTC​CCC​TCC​TTT​TGA​TGA
	Reverse	TCG​CCC​CTT​GCT​TGG​TAG​T
*Colocasia_esculenta_newGene_60403* (Starch synthase 4)	Forward	TTC​AGA​GCA​AAG​CAT​TAG​TGG​A
	Reverse	TTA​GTA​AGG​GAG​GGA​AGA​TCA​ACA
*Colocasia_esculenta_newGene_74347* (beta-amylase)	Forward	GGC​GAG​GGA​CCC​AAG​ATT​T
	Reverse	TGA​GCA​CCC​ACT​GTG​GTA​AGG
*Colocasia_esculenta_newGene_8502* (beta-fructofuranosidase)	Forward	CAC​CGT​GGA​ATG​GCT​GTC​T
	Reverse	GAG​GTC​TCC​ATC​CCG​TAG​TTG
*Colocasia_esculenta_newGene_92065* (glucan endo-1,3-beta-glucosidase)	Forward	GGA​AAT​GCA​AAT​AGA​TGG​AGC​C
	Reverse	TTC​GTA​GCA​ATG​TAA​TTG​TCG​G
*Colocasia_esculenta_newGene_93013* (beta-glucosidase)	Forward	CCA​CAG​ATA​CAA​GGA​AGA​TGT​TGA
	Reverse	AGC​CTG​TTG​TAA​TAT​GCC​ACT​C

### Data Analyses

The paired-end raw reads were processed as reported earlier (Fu et al., 2019). Briefly, the Quality Score (probability of base calling errors) were computed (Ewing et al., 1998) followed by base type distribution check. HISAT2 was used for the comparison of sequencing reads and read alignments, and StringTie was used to assemble the reads on the comparison pair. We used the taro genome [*C. esculenta* (Niue 2), https://db.cngb.org/search/project/PRJNA328799/] as reference sequence for alignment and subsequent analyses. The identified genes were functionally annotated in different data bases i.e., NR, Swiss-Prot, COG, KEGG, pfam, KOG, and GO as reported earlier (L. Chen et al., 2019). The gene expression was quantified using StringTie and expressed as Fragments Per Kilobase of transcript per Million fragments mapped (FPKM). Overall gene expression was represented as box plot. The read counts were used to determine the differential expression of genes. The genes/transcript with fold change >2 and false discovery rate (FDR) <0.05 were considered as differentially expressed genes (DEGs). Further we performed the KEGG pathway enrichment analysis for the DEGs and displayed the top 20 pathways with the most reliable enrichment significance (lowest Q-values) as a bubble chart. Then we manually selected the DEGs that were enriched in the pathways of interest and arranged their log 2 fold change values according to the selected taro corm age comparisons followed by the preparation of the heatmaps in TBtools (C. Chen et al., 2020).

For the data analysis of the small RNAs, we calculated the base quality value of the reads as reported earlier (Ewing and Green, 1998). We then removed the sequences smaller than 18 nt and greater than 30 nt, removed reads with low quality, and removed the reads with unknown N bases. The quality statistics of the miRNA sequencing were than represented as a table in Microsoft Excel 2019. The resulting sequences were then compared with the GtRNAdb, Rfam, and Repbase databases using Bowtie (Langmead et al., 2009). Sequence alignment was performed and filtered the rRNA, tRNA, snRNA, snoRNA, and other ncRNAs to obtain unannotated reads containing miRNAs. Bowtie was used to compare the sequences of unannotated reads with the reference genome to get the mapped reads. Furthermore, we compared the reads with the mature sequence of the known miRNAs in miRbase (v22). To predict the miRNAs that haven’t been previously reported, we used Biomark (which uses miRDeep2 software) (Friedländer et al., 2012). The expression of the miRNAs was quantified as transcripts per million (TPM). For detection of the differentially expressed miRNAs (DEmiRNAs), we used a screening criterion of log2 foldchange ≥0.58 and *p*-value ≤ 0.05 between the two samples. Further, we predicted the target genes for the known and newly identified miRNA in plants by using TargetFinder software (Allen et al., 2005). The annotation and KEGG pathway enrichment of the target genes of the miRNAs was done as reported above in the case of mRNA.

After determining the quality parameter of the RNA sequencing libraries, we used find_circ software to predict CircRNAs based on the following criteria. GU/AG appears on the both sides of the splice site, a clear breakpoint could be detected, have only two mismatches, the breakpoint should not appear outside the anchor two nucleotides, at least two reads support this junction, and the position of a short sequence that is aligned to the correct position is 35 points higher than that of other points. Furthermore, we calculated the distribution of CircRNA length in each sample (exon, intergenic region, and intron). We then predicted the positions of the CircRNA and its source genes on the reference genome followed by the prediction of CircRNA-miRNA targeting relationship by using TargetFinder (Bo and Wang, 2005).

The expression of the CircRNAs was computed as SRPBM (reads per billion mapped reads). For the screening of the differentially expressed CircRNAs we used log 2 foldchange ≥1.5 and *p*-value < 0.05. The annotation and KEGG pathway enrichment of the differentially expressed CircRNAs was done as described above for mRNA and miRNAs. The Principal Component Analysis was performed in R.

## Results

### Morpho-Biochemical Analysis of Taro Corms

**FIGURE 1:**
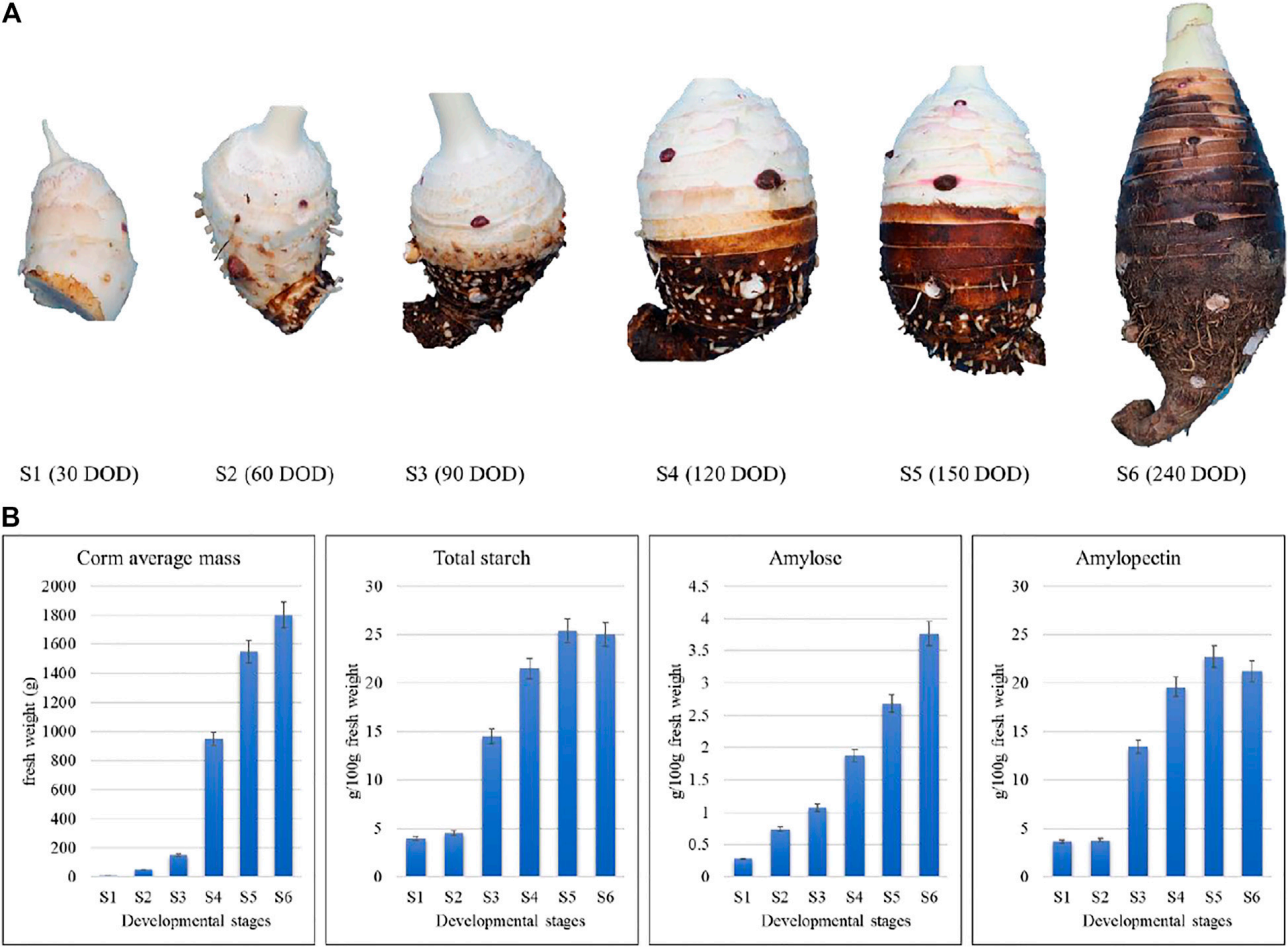
Morpho-biochemical comparison of taro corms based on growth stage and starch contents. S1, S2, S3, S4, S5, and S6 refer to the corm samples harvested after 1, 2, 3, 4, 5, and 8 months.

In this study, we sampled taro corms at six developmental stages, including S1, S2, S3, S4, S5, and S6 harvested after 1, 2, 3, 4, 5, and 8 months. The corms at the early developmental stages (S1 and S2) had very low amounts of starch, amylose and amylopectin as compared to later developmental stages (Figure 1A), indicating that thought the starch formation starts at early developmental stages but its accumulation in the corms increased rapidly after 2 months. Since there is a significant increase in starch contents at between S2 and S3 followed by a gradual increase in S4 and S5. Meanwhile, average mass of a corm significantly increases between S3 and S4 followed by gradual increase (Figure 1B).

### Taro Corm Transcriptome

**FIGURE 2:**
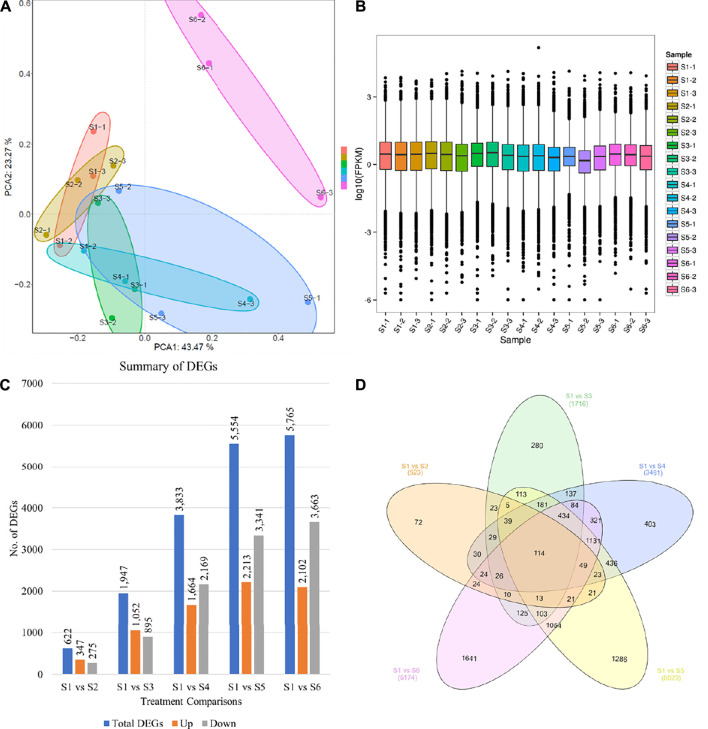
Taro corm transcriptome comparison statistics. **(A)** Principal component analysis of the genes that were differentially expressed between different treatments, **(B)** Overall distribution of gene expression (FPKM), **(C)** number of differentially expressed genes between the taro samples, and **(D)** Venn diagram representing the number of common and specific differentially expressed genes between the taro samples. S1, S2, S3, S4, S5, and S6 represent taro samples harvest after 1, 2, 3, 4, 5, and 8 months.

A total of 18 libraries (three biological replicates of each sample) were used to generate sequencing data. The data output statistics of each sample of this project are shown in Supplementary Table S1. After sequencing quality control, a total of 300.23 Gb clean data was obtained, and the percentage of Q30 bases in each sample was not less than 94.38%. The comparison efficiency between the Reads of each sample and the reference genome ranged from 88.88 to 90.65%. GC content ranged from 49.25 to 61.91%. ∼90% of the total reads could be mapped to the reference genome (Supplementary Table S1). The PCA showed that first and second principal components explained 43.47 and 23.27% variation, respectively (Figure 2A). Overall, the FPKM mean distribution of S3, S4, and S5 was lower than S1, S2, and S5 (Figure 2B). The comparison of five samples (S2 to S6) with S1 resulted in the identification of 622, 1,947, 3,833, 5,554, and 6,765 differentially expressed genes (DEGs), respectively (Figure 2C). Only 114 DEGs were common in all the five taro comparisons (Figure 2D).

The RNA-Seq data was validated through qRT-PCR. 10 transcripts were selected from starch and sucrose metabolism pathway for qRT-PCR (Supplementary Table S1; Supplementary Figure S1). The expression patterns of these DEGs were consistent with FPKM values of the same genes (Supplementary Tables S2, S3).

### Functional Annotation of DEGs

**FIGURE 3:**
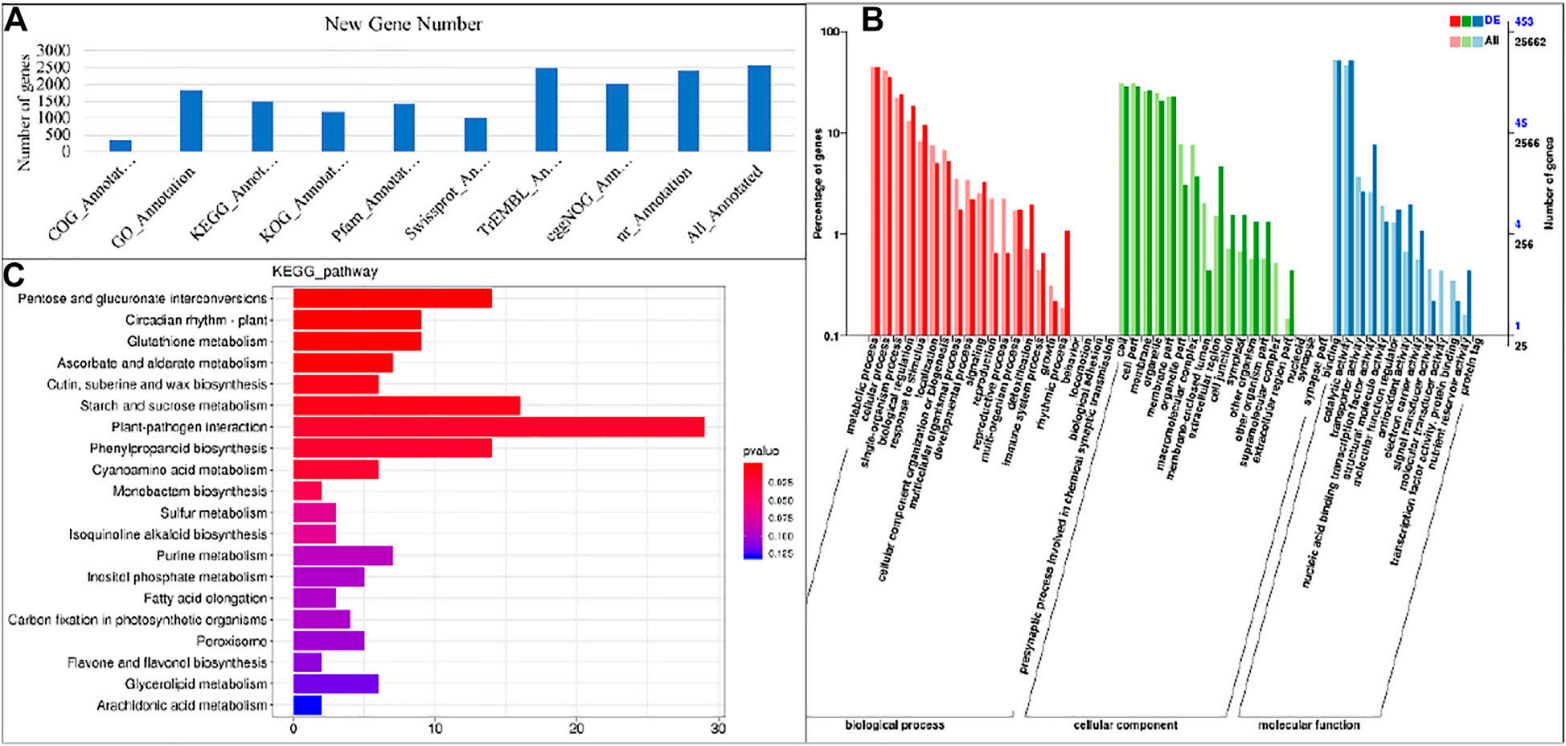
**(A)** Annotation summary of newly identified genes and Representation of gene’s enrichment in KEGG and GO pathways. **(A)** Gene ontology classification of assembled genes and **(B)** Histogram of enrichment of differentially expressed genes in KEGG pathways in S1 vs. S2.

Based on the selected reference genome sequence, StringTie software was used for mapping reads, and comparing with the original genome annotation information, finding the original unannotated transcription regions, discovering novel transcripts/genes, and to improve the original genome annotation information. The coded peptide chains having less than 50 amino acid residues or containing only a single exon sequence, were filtered out. A total of 11,203 genes were discovered and of which 2,558 were functionally annotated as new genes. This annotation was performed using the DIAMOND (Buchfink et al., 2015) software to compare the sequences with NR (Deng et al., 2006), Swiss-Prot (Apweiler et al., 2004), COG (Tatusov et al., 2000), KOG (Koonin et al., 2004), KEGG (Kanehisa, 2000), and to process the results for obtaining the new gene KEGG Orthology and other results. InterProScan (Jones et al., 2014) used the InterPro integrated database to analyze the GO Orthology results of the annotated genes (Ashburner et al., 2000). After predicting the amino acid sequence of the new gene, we used the HMMER (Eddy, 1998) software to compare with the Pfam (Punta et al., 2012) database to obtain the annotation information of the new gene. The final statistics on the number of new genes annotated by each database are shown in Figure 3A.

Based on the GO annotation, 1,824 new genes were grouped into three functional GO categories, i.e., Molecular Function (MF; 2,220), Cellular Component (CC; 1,295 sequences), and Biological Process (BP; 2,698 sequences), with subsets of sequences further divided into 11, 3, and 18 subcategories in these three groups, respectively (Figure 3B). There was a high representation of “binding” and “catalytic activity” in the category MF, which included 53.06 and 39.36% of the sequences in these subcategories, respectively. Furthermore, there was an enrichment of “cellular anatomical entity” (58.02%) and “intracellular” (33.75%) in the CC parental category, and a high representation of “cellular process” (37.69%), and “metabolic process” (36.21%) in the BP category.

### KEGG Pathways and Gene Ontology Enrichment Analysis

The significantly expressed DEGs were mapped on the KEGG pathways to identify the significantly enriched pathways. The DEGs were enriched in a total of twenty pathways. At different developmental stages of taro, highly enriched pathways included pentose and glucoronate interconversions pathway, carbon metabolisms, biosynthesis of amino acids and starch and sucrose metabolism pathway (Figure 3C). It is reported that the starch and sucrose metabolism pathway has a major contribution in the carbohydrate accumulation in different plant species (Zhu et al., 2017).

### Differential Regulation of Starch and Sucrose Metabolism and Pathways Present Both up and Downstream

**FIGURE 4:**
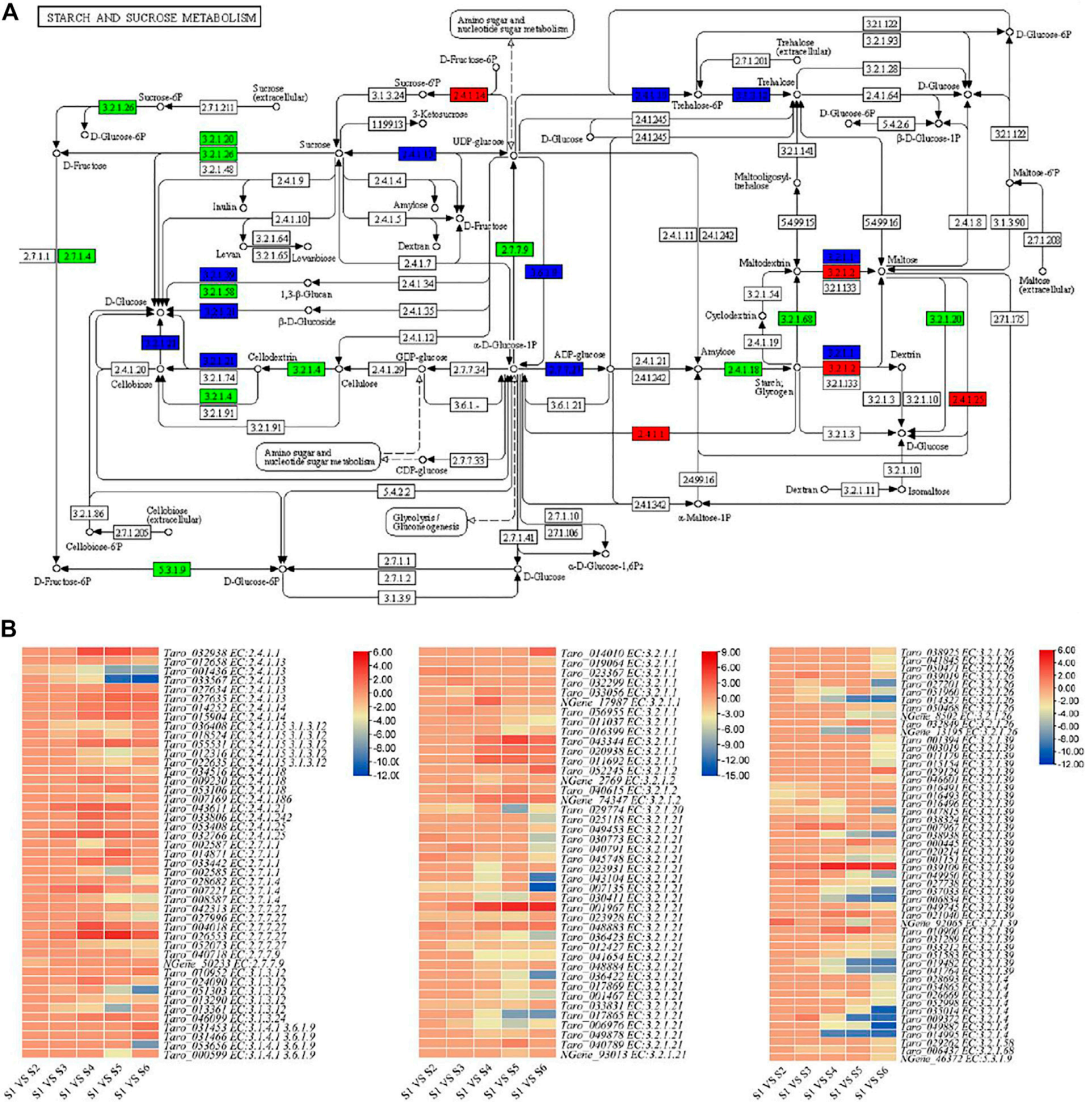
Enrichment of differentially expressed genes in starch and sucrose metabolism pathway. **(A)** Pathway map of starch and sucrose metabolism showing identified transcripts related to important enzymes of this pathway. Transcripts with elevated expression are shown in red. Downregulated transcripts are represented in green and transcript with both up- and downregulation are represented with blue color. **(B)** Heatmap representing log 2 foldchange values of the differentially expressed genes in starch and sucrose metabolism pathway.

A total of 139 DEGs associated with 24 important enzymes were enriched in starch and sucrose metabolism (Figure 4, Supplementary Table S2). These enzymes include sucrose synthetase [EC 2.4.1.13], ADP-glucose pyrophosphorylase [EC:2.7.7.27], beta-glucosidase [EC:3.2.1.21], Alpha-amylase [EC:3.2.1.1], beta amylase [EC:3.2.1.2], beta-glucosidase [EC:3.2.1.21] and glucan endo-1,3-beta-D-glucosidase [EC:3.2.1.39]. Most of the transcripts were differentially regulated in S4, S5 and S6. There were almost 32 transcripts related to beta-glucosidase [EC:3.2.1.21]. Sixteen of these 139 genes were only differentially expressed between S1 and S2; one alpha-amylase, three beta-glucosidases, and two glucan endo-1,3-beta-glucosidases were upregulated in S2 as compared to S1, while rest of the genes were downregulated. Actually, we found that different transcripts of the same genes were up- and downregulated, indicating that a possible interconversion of the intermediate products is going during these two stages. On the contrary, we found that 36, 77, 84, and 94 genes were differentially regulated between S1 vs. S3, S1 vs. S4, S1 vs. S5, and S1 vs. S6, respectively. These changes clearly indicate that large scale changes in the starch and sucrose metabolism occur during the fourth to eighth month of taro corm development. Apart from starch and sucrose metabolism, 87 and 140 genes were enriched in amino sugar and nucleotide sugar metabolism and glycolysis/gluconeogenesis pathways, respectively. Only six genes were differentially regulated between S1 and S2, whereas relatively higher number of genes were differentially regulated in S1 and stages latter than S3 i.e., S4, S5, and S5; 58 and 59 genes were differentially regulated in S1 vs. S5 and S1 vs. S6, respectively in amino sugar and nucleotide sugar metabolism pathway (Supplementary Table S2).

### The Prediction of New CircRNA

After performing the quality control of sequencing, a total of 300.21 Gb Clean Data was obtained, and the percentage of Q30 bases in each sample was not less than 97.94%, which indicated the use of high-quality clean reads in current study. From the statistics of the comparison results, the comparison efficiency of the Reads of each sample and the reference genome ranges from 99.74 to 99.94%. (Supplementary Table S1). It indicated that the sequence data is qualified for auxiliary analysis.

**FIGURE 5:**
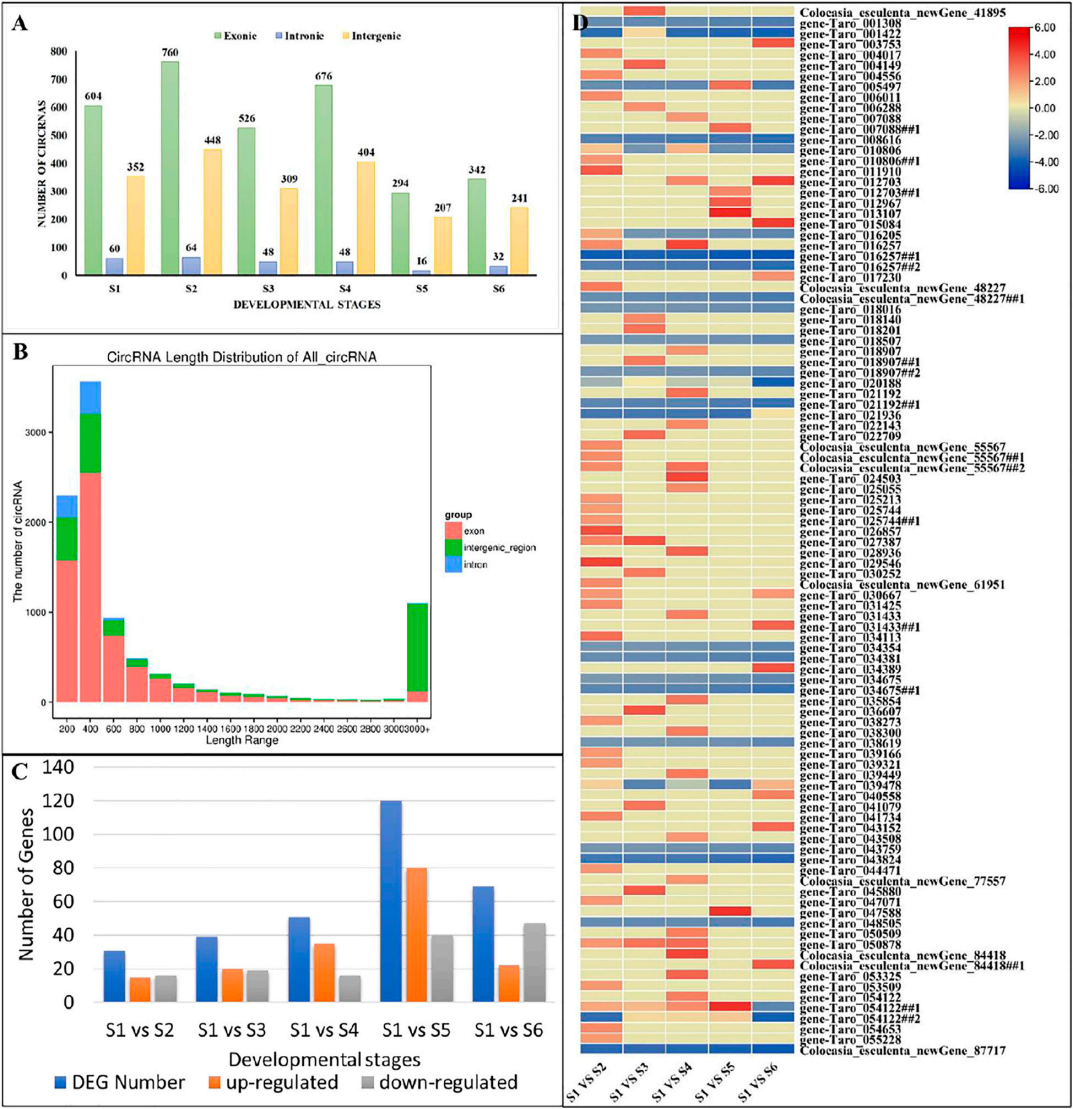
Characterization of CircRNAs. **(A)** Distribution of the identified CircRNAs accrsoos growth stages. **(B)** Length distribution of CircRNAs. **(C)** Numbers of differentially expressed CircRNAs in pairwise comparisons of growth stages. **(D)** expression patterns of differentially expressed CircRNAs in pairwise comparisons of growth stages. Genes with the symbols ##, represents spliced variants.

Based on the sequence reads, the number of candidate CircRNAs identified in the 18 samples ranged from 277 to 1,372 (Supplementary Table S3). In context of their origin, these CircRNAs are grouped into three categories as exonic, intronic and intergenic CircRNAs. Among the total 9,524 CircRNAs identified in *C. esculenta*, most were exonic CircRNAs (53–61%), followed by intergenic CircRNAs (33–41%) and intronic CircRNAs (1–6%) (Figure 5A). It is important to note that the length distribution of these CircRNAs ranged from 28 to 99,844 bp. The most abundant lengths were in the range from 200 to 600 bp, and the longest CircRNAs with more than 3,000 bp were generated from exonic and intergenic regions (Figure 5B).

To investigate the CircRNAs biological function during growth of taro corm, we compared the expression levels of CircRNAs among growth stages (S1 vs. S2, S1 vs. S3, S1 vs. S4, S1 vs. S5, and S1 vs. S6). A total of 191 CircRNAs were differentially expressed between the studied comparisons (Supplementary Table S3). Among them, 153 were known CircRNAs and 38 were newly identified. Remarkably, most CircRNAs seem to be specifically expressed between S1 and S5 (120 differentially expressed, 80 up regulated and 20 down regulated; Figure 5C; Supplementary Table S3). Almost 99 of the 191 CircRNAs were associated with those miRNAs (or target genes) that were enriched in starch and sucrose metabolism pathway (Figure 5D; Supplementary Table S3, see highlighted yellow cells).

### Identification of Micro RNA

**FIGURE 6:**
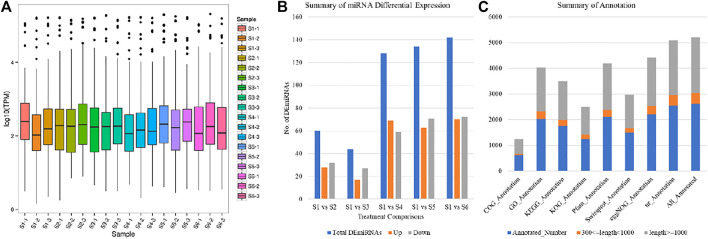
Characterization of miRNAs **(A)** Overall expression of miRNAs, **(B)** summary of differentially expressed miRNAs, and **(C)** annotation summary of the miRNA target genes in taro.

The Micro RNA (miRNA) sequencing of 18 taro samples resulted in a total of 375.10 M clean reads (average clean reads per sample were 15.50). The average Q30% and GC% was 97.29 and 47.1%, respectively. On an average 47.48% reads could be mapped on the reference genome (Supplementary Table S1). Based on these clean reads, we identified 205 miRNAs; 10 known and 195 newly predicted miRNAs. Overall transcript per million (TPM) of the miRNAs was variable between the different aged taro corms (Figure 6A). There were 60, 44, 128, 134, and 142 differentially expressed miRNAs (DEmiRNAs) detected between S1 vs. S2, S1 vs. S3, S1 vs. S4, S1 vs. S5, and S1 vs. S6, respectively (Figure 6B). The high number of DEmiRNAs between the S1 and the older stages i.e., S5 and S6 indicates that miRNAs might target a large number of genes during these age comparisons. The 10 known and 195 newly identified miRNAs were associated with 145 and 4,106 target genes; 2,613 of which could be annotated in different databases (Figure 6C).

The target genes of the miRNAs were significantly enriched in mRNA-surveillance pathway, plant hormone signal transduction, and RNA transport (Supplementary Figure S2). We also focused on the miRNA target genes that were enriched in starch and sucrose metabolism pathway. Of the predicted target genes, we found that 33 DEGs were enriched in starch and sucrose biosynthesis pathway. However, only four of these 33 genes were differentially expressed in the studied treatment comparisons. These 33 genes were target for 46 different miRNAs; 45 newly identified and one known miRNA (*aqc-miR156b*) (Table 2).

**TABLE 2:** List of genes and associated miRNAs that were enriched in starch and sucrose metabolism pathway.

Gene ID	Gene description	miRNA
*gene-Taro_019805*	alpha-amylase [EC:3.2.1.1]	novel_miR_105; novel_miR_49
*gene-Taro_006317*		novel_miR_12; novel_miR_147; novel_miR_162; novel_miR_169; novel_miR_198; novel_miR_210; novel_miR_48; novel_miR_75
*gene-Taro_006204*		novel_miR_112; novel_miR_94; novel_miR_98
*gene-Taro_007911*		novel_miR_179
*gene-Taro_029774*	alpha-glucosidase [EC 3.2.1.20]	novel_miR_111; novel_miR_126; novel_miR_201; novel_miR_22; novel_miR_43; novel_miR_15
*Colocasia_esculenta_newGene_2769*	beta-amylase [EC:3.2.1.2]	novel_miR_7
*gene-Taro_027220*	beta-fructofuranosidase [EC:3.2.1.26]	novel_miR_11
*gene-Taro_014327*		novel_miR_15
*gene-Taro_047897*		novel_miR_196
*gene-Taro_029867*		novel_miR_65
*gene-Taro_020067*		novel_miR_121
*gene-Taro_016898*		novel_miR_148
*gene-Taro_048354*		novel_miR_127; novel_miR_177; novel_miR_196; novel_miR_2; novel_miR_41; novel_miR_59; novel_miR_70
*gene-Taro_048263*		novel_miR_121
*gene-Taro_041504*	glucan endo-1,3-beta-glucosidase [EC:3.2.1.39]	novel_miR_127; novel_miR_177; novel_miR_2; novel_miR_41; novel_miR_59; novel_miR_70
*gene-Taro_028508*		novel_miR_193; novel_miR_87
*gene-Taro_049745*		novel_miR_125
*gene-Taro_051583*		novel_miR_112; novel_miR_94; novel_miR_98
*Colocasia_esculenta_newGene_73911*		aqc-miR156b
*gene-Taro_044097*		novel_miR_196
*gene-Taro_055611*		novel_miR_196
*gene-Taro_007421*		novel_miR_179
*gene-Taro_015719*		novel_miR_6
*gene-Taro_008604*		novel_miR_196
*gene-Taro_017902*		novel_miR_196
*gene-Taro_047489*		novel_miR_196
*gene-Taro_001951*		novel_miR_196
*gene-Taro_012457*		novel_miR_196
*gene-Taro_024129*	starch synthase [EC:2.4.1.21]	novel_miR_112; novel_miR_94; novel_miR_98
*gene-Taro_026046*	trehalose 6-phosphate phosphatase [EC:3.1.3.12]	novel_miR_156; novel_miR_188
*gene-Taro_023524*		novel_miR_15; novel_miR_6
*gene-Taro_018385*		novel_miR_179
*gene-Taro_021354*		novel_miR_175; novel_miR_186; novel_miR_197; novel_miR_29; novel_miR_32; novel_miR_76

**FIGURE 7:**
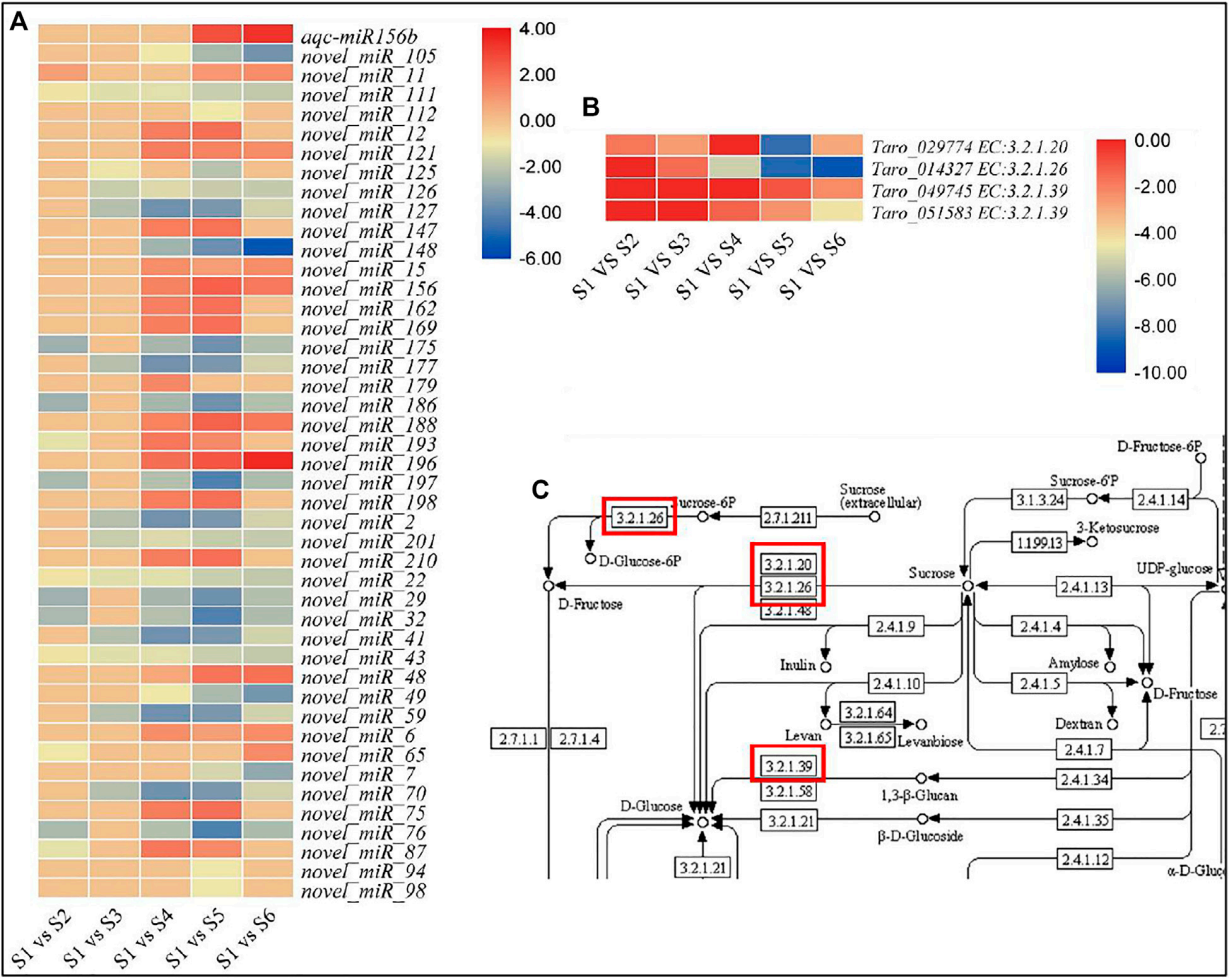
Expression profiles of miRNAs. **(A)** miRNAs expressed in pairwise comparisons of growth stages. **(B)** Expression profiles of selected miRNA target genes. **(C)** Starch and sucrose metabolisms pathway representing enzymes identified as the targets of selected miRNAs.

The expression of 18 of the newly identified miRNAs was reduced in at least one of the later stages i.e., S2, S3, S4, S5, and S6. The same number of miRNAs showed increased expressions in the studied stages as compared to S1. The remaining nine had variable expression patterns in different treatment comparisons (Figure 7).

## Discussion

### The Transcriptome of *C. esculenta* Corm During Development

Since the edible part of Taro is the corm, therefore, understanding the multitude of changes, especially during development is an important task of taro breeders (Boampong et al., 2020). The corm development starts as early as 2 weeks after planting followed by a rapid growth in the first 2 months in the rainfed areas whereas this growth is slightly delayed in irrigated conditions (up to 3–5 months). The maximum corm weight is reached in 10–11.5 months. However, farmers start harvesting the corms as early as 8 months after planting (Ahmadizadeh et al., 2011; Boampong et al., 2020). Therefore, we opted to study the transcriptome of taro corms of ages 1, 2, 3, 4, 5, and 8 months. The observations that the DEGs were significantly enriched in starch and sucrose metabolism, carbon metabolism, biosynthesis of amino acids, and pentose and glucoronate interconversions suggests that during corm development carbon metabolism plays an essential role in the carbon assimilation (Figure 2). Since starch is present in the corm in highest concentrations as compared to other nutrients (Temesgen and Retta, 2015), therefore, the regulation of starch and sucrose metabolism and associated pathways i.e., pentose and glucoronate interconversions proposes large scale changes in the starch and sucrose concentrations in the studied time points of corm development (Gao et al., 2018). This was further confirmed by the GO enrichment analysis where major portion of the genes were enriched in metabolic process, cellular process, and biological regulations. These large-scale changes in the growth and development of corm are also evident from the observation that 5,765 and 5,554 DEGs were regulated in S1 vs. S5 and S1 vs. S6, respectively (Figure 2). A similar trend for the differential expression of CircRNAs and miRNAs confirmed that at the later stages of corm development, significant transcriptomic changes take place (Figure 4C; Figure 5B). Together these processes are responsible for overall corm mass increase from 8.77 g/plant (S1) to 1800 g/plant (S6) (Figure 1). In this regard, the highly upregulated genes in S2 to S6 as compared to S1 are ideal candidates for future research. For example, *C. esculenta_newGene_69493, C. esculenta_newGene_47533,* and *C. esculenta_newGene_83404* are newly identified genes in this species which showed highest log 2 foldchange value in S2 as compared to S3. According to GO enrichment these are involved in nucleic acid binding (MF) (Schultz and Champoux, 2008; Vicient and Casacuberta, 2020). A detailed understanding of these genes will elaborate their roles in early corm development.

### Starch and Sucrose Metabolism Significantly Contributes to Variations in Tarostarch Content

Starch is one of the most abundant compounds found in the corm (storage organ) of *C. esculenta*. It is already known that starch accumulation and enlargement of storage organ is a parallel process (Burton, 1978). Therefore, a higher coordination exists between storage organ formation and starch synthesis (Geigenberger et al., 1994). The observation that starch content significantly increased up to S5 as compared to S1 is possibly due to the increased expression of genes encoding beta-glucosidase [EC:3.2.1.21], ADP-glucose pyrophosphorylase [EC:2.7.7.27], Alpha-amylase [EC:3.2.1.1], beta amylase [EC:3.2.1.2], beta-glucosidase [EC:3.2.1.21], and glucan endo-1,3-beta-D-glucosidase [EC:3.2.1.39] and granule-bound starch synthase [EC:2.4.1.242] (Figure 3). Granule-bound starch synthase is a major contributor towards starch synthesis in storage organs of plants (Nakamura et al., 1998). There are two types of granule-bound starch synthase (GBSSI and GBSSII) in plants and have different expression profiles. In transgenic rice, GBSSI positively affected the content of amylose. Moreover, the difference in amount of amylose in transgenic and non-transgenic plants resulted from long unit chains of amylopectin (Hanashiro et al., 2008). The DEGs identified in current study belonged to both GBSSI EC 2.4.1.21 (*gene_Taro_043611*), and GBSSII EC:2.4.1.242 (*gene_Taro_033806*). Both transcripts were differentially upregulated in all growth stages with higher expression in S3, S4 and S5. Additionally, there was a lack of correlation between starch contents and the expression of important starch degradation enzymes including sucrose-phosphate synthase [EC:2.4.1.14], disproportionating enzyme [EC:2.4.1.25], and alpha/beta amylase [EC:3.2.1]. It indicates that the gene expression analysis of starch synthesizing or degradation enzymes is not enough to decide the ultimate factors responsible for the variation of starch contents in corms (K. Zhang et al., 2017). The expression of genes encoding key enzymes of starch and sucrose metabolism pathway (GBSS, AGPase, SP, SSS and SuSy) demonstrated significant variations at stage S4. It is in accordance to starch accumulation in corms which almost peaked at S4, indicating that S4 is potentially the most important stage in starch biosynthesis (Figure 1).

The structure and important features of starch are significantly affected by amylose contents and amylose to amylopectin ratio. In sweet potato, RNA interference was used to suppress the expression of GBSSI and SBEII to produce amylose-free and high-amylose transgenic plants, respectively (Shimada et al., 2006; Kitahara et al., 2007; Otani et al., 2007). It affirms a critically important role of these enzymes in controlling starch composition. In current analysis, the two GBSS encoding genes (*gene_Taro_043611* and *gene_Taro_033806*) were expressed at relatively higher levels at S4 and S5, while amylose to amylopectin ratio was still increasing. Similarly, expression of *gene_Taro_004018* and *gene_Taro_026553* (AGPase) was significantly upregulated at S4. It indicates that variation in the expression of these genes may potentially affect the variation in starch composition. However, the expression of genes encoding other starch-synthesizing enzymes, including SBE [EC 2.4.1.18], and ISA [EC:3.2.1.68], was not directly correlated with the composition of starch in corms. It is reported that the properties of starch are dependent on a coordinated expression of all the genes in a pathway and not on a singular gene product (Lai et al., 2016). Since the synthesis of amylose and amylopectin follows a multifaceted procedures including several starch synthesizing enzymes (Zeeman et al., 2010; Lai et al., 2016), we may conclude that the transcript abundance on an individual starch-synthesizing enzyme would not be enough to decide starch composition in corm.

Accumulation of starch in a storage organ is a continuous activity that involves the synthesis, transport, degradation, and inter-conversion of starch and sucrose (Zeeman et al., 2010; Schreiber et al., 2014). The cleaved products of sucrose (major photo-assimilate) are used in plant storage organs to synthesis starch (X.-Q. Li and Zhang, 2003). The enzymes affecting metabolism and/or cleavage of sucrose potentially play key role in starch accumulation. There are two ways for sucrose cleavage in the cytosol; 1) beta-fructofuranosidase [EC:3.2.1.26] mediated conversion of sucrose into fructose and glucose, and ii), invertase or sucrose synthetase [EC 2.4.1.13] converts sucrose into fructose and UDP-glucose (X.-Q. Li and Zhang, 2003; Wind et al., 2010). Later on, UGPase [EC:2.7.7.9] converts the UDP-glucose into glucose-1-phosphate, which is used in subsequent starch synthesis. In current study, 5 sucrose synthetase, 2 UGPase and 11 beta-fructofuranosidase encoding differentially expressed transcripts were detected, and most of these unigenes were expressed during all developmental stages examined, indicating that these genes have essential roles in corms.

### Possible Roles of CircRNA and miRNAs in Corm Development and Starch and Sucrose Metabolism

CircRNAs function as miRNA sponges and have been studied for their participation in miRNA-related pathways where they might regulate genes expression (P. Zhang et al., 2020). The 191 differentially expressed CircRNAs could be associated with the corm development in taro. We propose this because these CircRNAs (or their target genes) were enriched in amino acid biosynthesis and protein processing in endoplasmic reticulum, RNA transport, starch and sucrose metabolism, plant hormone signal transduction, and carbon metabolism. Particularly, the amino acid biosynthesis and carbon metabolism pathways are significantly important for early corm development since they directly impact nitrogen use efficiency and carbon partitioning in source-sink tissues (Hajirezaei et al., 2000; Dellero, 2020). In this regard, the 99 of the 191 CircRNAs are good candidates for their roles in the regulation of miRNAs and their target genes in starch and sucrose metabolism. Of the known miRNAs, aqc-miR156b has been previously reported in Colorado blue columbine (*Aquilegia coerulea*) (Puzey and Kramer, 2009). However, its functional validation is still to be done but it has been reported that its expression increased in clubroot infected *Brassica napus* L. plants 10 days after infection suggesting its role in either stress response or establishment of clubroot (Verma et al., 2014). In our experiment, this miRNA didn’t differentially express between S1 and S2, S3, and S4. Its expression increased in S5 (log 2 fold change = 2.67) and S6 (log 2 fold change = 3.30) as compared to S1. Its target gene was a glucan endo-1,3-beta-glucosidase 5/6 (*gene-Taro_049745* and *gene-Taro_051583*). Most of the DEGs annotated as glucan endo-1,3-beta-glucosidase were downregulated in the later growth stages (S5 and S6) as compared to S1 except *gene-Taro_001976* (which is upregulated S4, S5 and S6). Thus, there could be negative relationship between the expression of aqc-miR156b and glucan endo-1,3-beta-glucosidase 5/6; it converts 1,3-β-glucan into D-glucose (Reese and Mandels, 1959). However, there were other novel miRNAs (*novel_miR94, novel_miR98, novel_miR112, and novel_miR125*) that were also associated with this enzyme. Future studies would help to reveal the possible role of these miRNAs in related to this enzyme.

Other than the two glucan endo-1,3-beta-glucosidases, the differential expression of an alpha-glucosidase (*gene-Taro_029774*) and a beta-fructofuranosidase (*gene-Taro_014327*) between different treatment comparisons (Figure 6) is important. The alpha-glucosidase converts sucrose into D-fructose (Lebosada and Librando, 2017) whereas beta-fructofuranosidase also serves the same purpose in plants (Lopez et al., 1988). We found that these genes are the targets of novel_miR_22, novel_miR_43, novel_miR_126, novel_miR_201 (alpha-glucosidase) and novel_miR_15 (beta-fructofuranosidase). Both the genes were downregulated in other growth stages as compared to S1 (Figure 6). The transcript abundances of the miRNAs associated with the alpha-glucosidase were also decreased in all treatments (negative log 2 fold change values) as compared to S1. While that of novel_miR_15 was increased in all taro corms (S2-S6) as compared to S1. These results suggest that the hydrolysis of the sucrose into D-fructose might be affected by the targeted differential changes in the abundances of these miRNAs to the alpha-glucosidase and fructofuranosidase.

## Conclusion

Current study reported the variations in starch accumulation and the differential expression of mRNAs, CircRNAs and miRNAs in six different growth stages of Taro corm development. A potential correlation starch/sucrose metabolism pathway and gene expression was also discussed. Although some of these genes were already reported, a large number of reported coding and non-coding genes were reported for the first time Taro corm. This study revealed important candidates involved in the biosynthesis and metabolism of starch and sugars during corm formation and growth. The information generated from current research will be a valuable foundation for deciphering molecular and physiological mechanisms governing starch and sucrose properties of Taro corms.

## Data Availability

The datasets presented in this study can be found in online repositories. The names of the repository/repositories and accession number(s) can be found in the article/Supplementary Material.

## Abbreviations

mRNA, messenger RNA; miRNA, micro RNA; CircRNA, circular RNA; NR, non redundant sequence database; COG, orthologous groups of proteins database; KEGG, kyoto encyclopedia of genes and genomes database; Pfam, protein families database; KOG, euKaryotic orthologous groups database; GO, the gene ontology knowledgebase.
