# Supplementary material for: Comparative Transcriptome Sequencing of Taro Corm Development With a Focus on the Starch and Sucrose Metabolism Pathway
Source: Front Genet. 2021 Oct 22;12:771081. doi: 10.3389/fgene.2021.771081 (PMC8630585; doi:10.3389/fgene.2021.771081)
Supplement: Supplementary file 1 [file Presentation1.PPTX]

## Slide 1
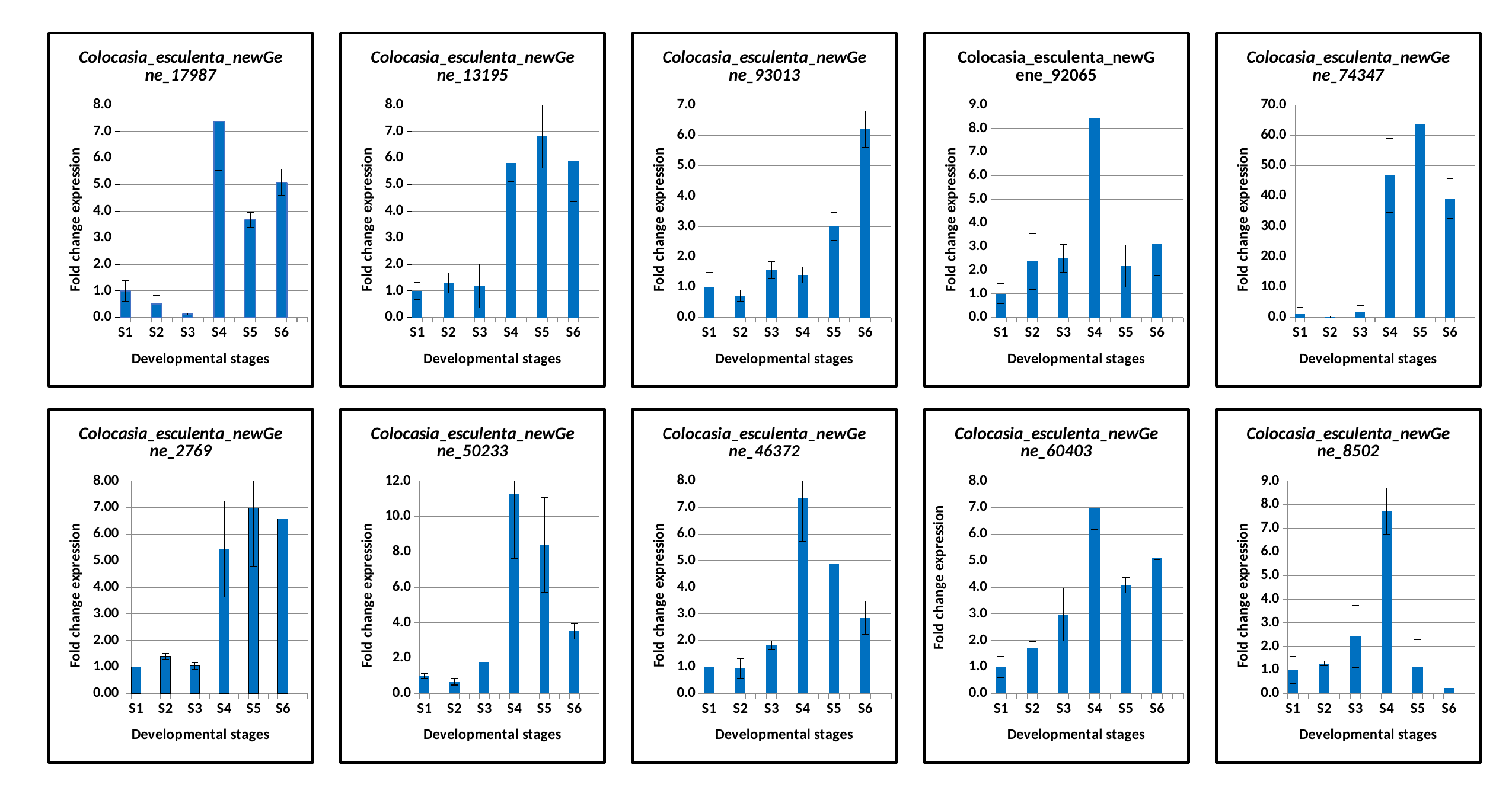

### Chart: Colocasia_esculenta_newGene_17987
| Category | |
|---|---|
| S1 | 1.0 |
| | None |
| | None |
| S2 | 0.49535948361485643 |
| | None |
| | None |
| S3 | 0.12337606508932876 |
| | None |
| | None |
| S4 | 7.37931522024878 |
| | None |
| | None |
| S5 | 3.6753344920793474 |
| | None |
| | None |
| S6 | 5.085462155249329 |
### Chart: Colocasia_esculenta_newGene_13195
| Category | |
|---|---|
| S1 | 1.0 |
| | None |
| | None |
| S2 | 1.300584377973593 |
| | None |
| | None |
| S3 | 1.1920997833549554 |
| | None |
| | None |
| S4 | 5.8038457079797 |
| | None |
| | None |
| S5 | 6.8195033122733415 |
| | None |
| | None |
| S6 | 5.877855937959217 |
### Chart: Colocasia_esculenta_newGene_93013
| Category | |
|---|---|
| S1 | 1.0 |
| | None |
| | None |
| S2 | 0.71498505929403 |
| | None |
| | None |
| S3 | 1.5626521498473571 |
| | None |
| | None |
| S4 | 1.399845396486767 |
| | None |
| | None |
| S5 | 2.9995350483005137 |
| | None |
| | None |
| S6 | 6.20245773805579 |
### Chart: Colocasia_esculenta_newGene_92065
| Category | |
|---|---|
| S1 | 1.0 |
| | None |
| | None |
| S2 | 2.3686955464342736 |
| | None |
| | None |
| S3 | 2.502975977024286 |
| | None |
| | None |
| S4 | 8.46125664307569 |
| | None |
| | None |
| S5 | 2.173824626574911 |
| | None |
| | None |
| S6 | 3.094588921714108 |
### Chart: Colocasia_esculenta_newGene_74347
| Category | |
|---|---|
| S1 | 1.0 |
| | None |
| | None |
| S2 | 0.13464896075366736 |
| | None |
| | None |
| S3 | 1.6524080572506805 |
| | None |
| | None |
| S4 | 46.85970066514998 |
| | None |
| | None |
| S5 | 63.6639848429093 |
| | None |
| | None |
| S6 | 39.15550726553771 |
### Chart: Colocasia_esculenta_newGene_2769
| Category | |
|---|---|
| S1 | 1.0 |
| | None |
| | None |
| S2 | 1.4087347569806021 |
| | None |
| | None |
| S3 | 1.0476958805569476 |
| | None |
| | None |
| S4 | 5.4406694013808865 |
| | None |
| | None |
| S5 | 6.982923226310564 |
| | None |
| | None |
| S6 | 6.587616938659691 |
### Chart: Colocasia_esculenta_newGene_50233
| Category | |
|---|---|
| S1 | 1.0 |
| | None |
| | None |
| S2 | 0.666708642233918 |
| | None |
| | None |
| S3 | 1.7983770820714977 |
| | None |
| | None |
| S4 | 11.260553462633371 |
| | None |
| | None |
| S5 | 8.402061427045378 |
| | None |
| | None |
| S6 | 3.5127477974314187 |
### Chart: Colocasia_esculenta_newGene_46372
| Category | |
|---|---|
| S1 | 1.0 |
| | None |
| | None |
| S2 | 0.9351102232134035 |
| | None |
| | None |
| S3 | 1.8155038631450253 |
| | None |
| | None |
| S4 | 7.363870044648295 |
| | None |
| | None |
| S5 | 4.861858069100896 |
| | None |
| | None |
| S6 | 2.839659164257535 |
### Chart: Colocasia_esculenta_newGene_60403
| Category | |
|---|---|
| S1 | 1.0 |
| | None |
| | None |
| S2 | 1.7026002203069035 |
| | None |
| | None |
| S3 | 2.9839074265554486 |
| | None |
| | None |
| S4 | 6.980408123217233 |
| | None |
| | None |
| S5 | 4.084050075521694 |
| | None |
| | None |
| S6 | 5.107623638553757 |
### Chart: Colocasia_esculenta_newGene_8502
| Category | |
|---|---|
| S1 | 1.0 |
| | None |
| | None |
| S2 | 1.2714551350521537 |
| | None |
| | None |
| S3 | 2.4172927813495715 |
| | None |
| | None |
| S4 | 7.739085270499456 |
| | None |
| | None |
| S5 | 1.1132698481125876 |
| | None |
| | None |
| S6 | 0.23280185264888983 |
