# Supplementary figures and images for: Comparative Transcriptome Sequencing of Taro Corm Development With a Focus on the Starch and Sucrose Metabolism Pathway
Source: Front Genet. 2021 Oct 22;12:771081. doi: 10.3389/fgene.2021.771081 (PMC8630585; doi:10.3389/fgene.2021.771081)

## Slide 1
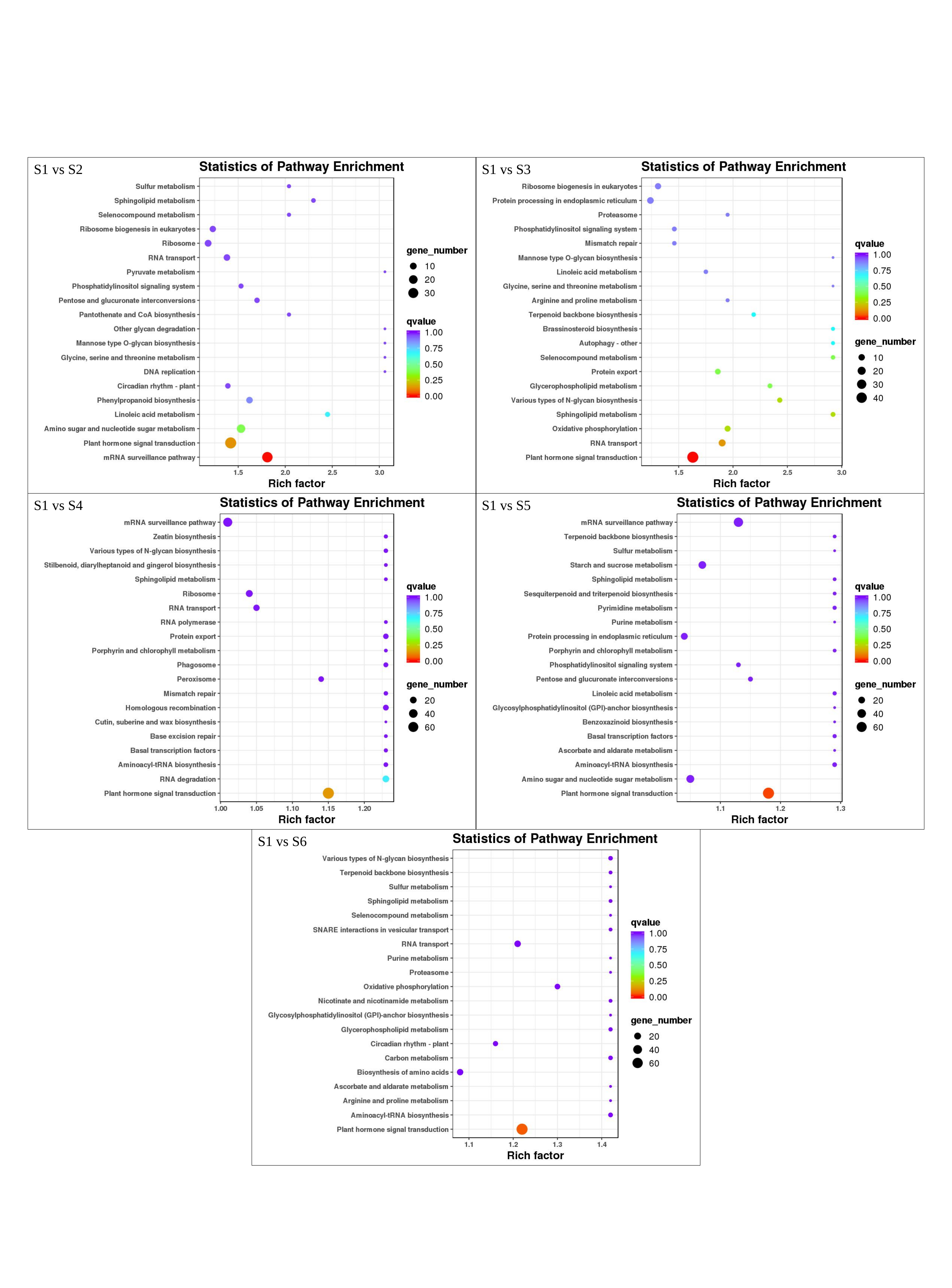

S1 vs S2
S1 vs S3
S1 vs S4
S1 vs S5
S1 vs S6

Supplement: Supplementary file 2 [file Presentation2.PPTX]
